# Navigating the Hemostatic Balance: Anticoagulation and Antiplatelet Therapy in Patients with Thrombocytopenia

**DOI:** 10.3390/jcm15062273

**Published:** 2026-03-17

**Authors:** María-Eva Mingot-Castellano, María Teresa Álvarez Román, Jose María Bastida, Nora Butta, Gonzalo Caballero Navarro, Mariana Canaro Hirnyk, Laura Entrena Ureña, Maria del Carmen Gómez del Castillo Solano, Andres Ramirez Lopez, Blanca Sánchez González, David Valcarcel Ferreira, Cristina Pascual Izquierdo

**Affiliations:** 1 Hematology Department, Molecular Diagnostics Department, Hospital Universitario Virgen del Rocío, Instituto de Biomedicina de Sevilla (IBIS/CSIC), Universidad de Sevilla, 41013 Sevilla, Spain; 2 Hematology Department, La Paz University Hospital-IdiPaz, Universidad Autónoma de Madrid, 28049 Madrid, Spain; 3 Hematology Department, Complejo Asistencial Universitario de Salamanca (CAUSA), Instituto de Investigación Biomédica de Salamanca (IBSAL), Universidad de Salamanca (USAL), 37008 Salamanca, Spain; jmbastida@saludcastillayleon.es; 4 Bleeding and Haemostasis Disorders Group-IdiPAZ, Haematology and Haemotherapy Department, La Paz University Hospital, 28046 Madrid, Spain; nora.butta@salud.madrid.org; 5 Hematology Department, Hospital Universitario Miguel Servet, 50009 Zaragoza, Spain; gcaballeron@salud.aragon.es; 6 Hematology Department, Hospital Universitario Son Espases, 07120 Palma de Mallorca, Spain; 7 Hematology Department, Hospital Universitario Virgen de las Nieves, 18014 Granada, Spain; 8 Hematology Department, Hospital Universitario Lucus Augusti, 27003 Lugo, Spain; 9 Hematology Department, Hospital Universitario de Toledo, 45007 Toledo, Spain; andres.ramirez1793@gmail.com; 10 Hematology Department, Hopital Parc de Salut Mar, 08003 Barcelona, Spain; 11 Hematology Department, Hospital Universitario Vall d’Hebron, 08035 Barcelona, Spain; 12 Hematology Department, Hospital General Universitario Gregorio Marañon, Instituto de Investigación Gregorio Marañon, 28040 Madrid, Spain

**Keywords:** thrombocytopenia, thrombosis, stroke, ischemia, anticoagulant, antiplatelet, heparin, DOAC, bleeding, cancer, ITP, TTP, TMA, cirrhosis

## Abstract

**Background:** Thrombocytopenia is traditionally perceived as a bleeding-predominant condition; however, growing evidence indicates that many thrombocytopenic states are paradoxically associated with an increased risk of venous and arterial thrombosis. This dual hemostatic derangement poses major therapeutic challenges when anticoagulant or antiplatelet therapy is indicated, particularly in complex settings such as cancer-associated thrombosis, immune thrombocytopenia, and advanced liver disease. **Methods:** We conducted a narrative review of the literature published between January 2021 and May 2025 using PubMed and guideline repositories. Search terms included thrombocytopenia, anticoagulation, antiplatelet therapy, cancer-associated thrombosis, immune thrombocytopenia, and cirrhosis. International guidelines from ASH, ISTH, ASCO, EHA, ESC, and AHA were prioritized. Evidence was synthesized to define platelet-based safety thresholds and disease-specific management strategies. **Results/Discussion:** Thrombocytopenia does not uniformly confer protection against thrombosis. Platelet activation, endothelial dysfunction, inflammatory signaling, impaired fibrinolysis, and procoagulant microparticles contribute to a prothrombotic milieu despite reduced platelet counts. Most guidelines support full-dose anticoagulation at platelet counts ≥ 50 × 10^9^/L, with dose modification between 25 and 50 × 10^9^/L and treatment interruption below 25 × 10^9^/L, depending on thrombotic risk. Antiplatelet therapy requires stricter individualization, particularly regarding dual antiplatelet therapy. **Conclusions:** Management of antithrombotic therapy in thrombocytopenic patients requires a dynamic, individualized approach balancing ischemic and bleeding risks. Pragmatic algorithms may guide clinical decision-making while prospective data remain limited.

## 1. Introduction

Thrombocytopenia is frequently encountered across a wide range of clinical contexts, including cancer-associated thrombosis (CAT), immune thrombocytopenia (ITP), chronic liver disease, infection, and critical illness. Its prevalence varies according to etiology, affecting up to 50% of patients receiving cytotoxic chemotherapy and a substantial proportion of those with cirrhosis or systemic autoimmune disorders [1,2,3]. Historically, thrombocytopenia has been regarded as a surrogate marker of bleeding risk, often prompting avoidance, dose reduction, or discontinuation of antithrombotic therapy, even in the presence of compelling thrombotic indications.

Over the past decade, this traditional paradigm has been increasingly challenged. Accumulating epidemiological and mechanistic evidence demonstrates that thrombocytopenia does not confer intrinsic protection against thrombosis and may, in several disease states, coexist with or even reflect a prothrombotic milieu [4]. Cancer-associated thrombosis frequently develops in patients with chemotherapy-induced thrombocytopenia [2,5], while patients with ITP exhibit a 1.5–2.5-fold increased risk of venous and arterial thromboembolic events compared with the general population [5,6,7,8]. Similarly, advanced liver disease is now recognized as a condition of “rebalanced” but fragile hemostasis, rather than a naturally anticoagulated state [3].

This coexistence of bleeding and thrombotic risk represents a true clinical paradox. The challenge is further compounded by the systematic exclusion of thrombocytopenic patients from pivotal trials evaluating anticoagulants and antiplatelet agents, including studies of antivitamin K, low-molecular-weight heparins (LMWHs), direct oral anticoagulants (DOACs), aspirin, and P2Y12 receptor antagonists [9]. As a result, available evidence is largely derived from retrospective cohorts, small observational studies, and expert consensus, leading to substantial heterogeneity in clinical practice.

Current international guidelines from societies such as the American Society of Hematology (ASH), the International Society on Thrombosis and Haemostasis (ISTH), and the American Society of Clinical Oncology (ASCO) provide overarching principles but limited platelet-count-specific recommendations [2,6,7,8]. Thresholds for full-dose anticoagulation, dose reduction, or treatment interruption vary widely, commonly ranging from 50 × 10^9^/L to 25 × 10^9^/L, reflecting the absence of prospective data [2,10]. Similarly, the use of platelet transfusions to “enable” anticoagulation remains poorly standardized, with uncertainty regarding optimal transfusion triggers and the balance between thrombotic, bleeding, and immunologic risks [2,6,8,9].

Evidence guiding antiplatelet therapy in thrombocytopenic patients is even more limited. Decisions regarding aspirin, clopidogrel, or dual antiplatelet therapy in contexts such as acute coronary syndromes, percutaneous coronary intervention, or structural heart interventions are largely opinion-based and rarely account for the underlying etiology of thrombocytopenia, whether immune-mediated, liver-related, or chemotherapy-induced [9,10].

The objective of this review is to synthesize current evidence on anticoagulation and antiplatelet therapy in thrombocytopenic patients, focusing on (i) the pathophysiological mechanisms underlying thrombosis at low platelet counts, (ii) disease-specific considerations in cancer-associated thrombosis, ITP, and liver disease, and (iii) evidence-based platelet thresholds to guide safe and effective antithrombotic therapy. Finally, we aim to provide a practical framework to support individualized clinical decisions in daily practice.

## 2. Review Methodology

We conducted a narrative review to summarize available evidence and provide pragmatic recommendations on antithrombotic management in thrombocytopenia. A structured literature search was performed in PubMed and on major professional society websites for guidelines/position papers (ASH, ISTH, ASCO, EHA, ESC, and AHA) from January 2021 to May 2025. Search terms combined thrombocytopenia with antithrombotic therapies (anticoagulation, heparin/LMWH, DOAC, warfarin, antiplatelet therapy, aspirin, P2Y12 inhibitors, and DAPT) and key clinical settings (cancer-associated thrombosis, immune thrombocytopenia, cirrhosis/liver disease, atrial fibrillation, acute coronary syndrome, PCI/stent, and thrombotic microangiopathy). Reference lists of key guidelines and reviews were hand-searched to capture additional relevant studies. We included: (i) international guidelines, consensus statements, and position papers; (ii) randomized trials, systematic reviews/meta-analyses, and prospective/retrospective cohort studies reporting clinical outcomes (bleeding, thrombosis recurrence, mortality) or platelet-count-adapted strategies; and (iii) selected mechanistic studies when directly informing clinical interpretation. We excluded isolated case reports, very small case series with limited generalizability, studies not addressing thrombocytopenia-specific management, and non-original commentary without supporting primary data. Two reviewers independently screened titles/abstracts and full texts; disagreements were resolved by consensus. Evidence was prioritized as: (1) international guidelines; (2) randomized trials; (3) meta-analyses; (4) large observational cohorts/registries; and (5) smaller observational studies, mainly for underrepresented scenarios. No full formal grading was undertaken; however, key studies and meta-analyses underwent a simplified appraisal (risk of confounding/selection and outcome ascertainment for observational studies; heterogeneity and publication-bias reporting for meta-analyses), which informed the strength and caution of the narrative synthesis.

## 3. Pathophysiological Basis of Thrombosis in Thrombocytopenia

**Figure 1:**
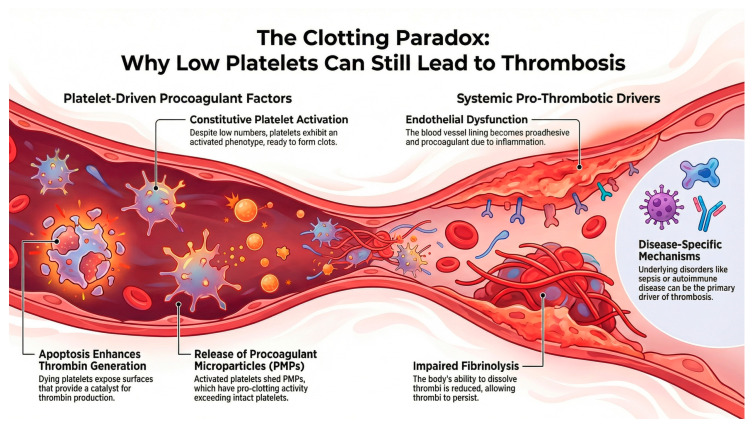
Clotting paradox in patients with thrombocytopenia. Coexistence of bleeding and thrombotic risk is observed in these patients. Thrombocytopenia represents a clinical paradox, with multiple mechanisms explaining this situation, as illustrated in the figure. Figure was generated by the authors using NotebookLM (Google LLC, Mountain View, CA, USA).

Thrombosis risk at low platelet counts is determined not only by platelet number but also by the interplay of platelet function, endothelial integrity, coagulation factors, fibrinolysis, and inflammation. Across thrombocytopenic states, qualitative platelet hyperreactivity, endothelial activation, and an imbalance between procoagulant and anticoagulant pathways can preserve or even enhance thrombin generation (Figure 1), supporting the concept of a fragile “rebalanced hemostasis” that may tilt toward bleeding or thrombosis depending on clinical triggers [11].

Several thrombocytopenic disorders show increased platelet activation markers and phosphatidylserine exposure, creating procoagulant surfaces that accelerate thrombin generation despite reduced platelet counts [12,13,14,15,16,17]. Activated or apoptotic cells release phospholipid-rich microparticles that can markedly amplify coagulation. Platelet-derived microparticles are often increased in immune-mediated and inflammatory thrombocytopenias and may correlate inversely with platelet count, contributing to ongoing thrombin generation [18,19,20]. Endothelial activation (e.g., increased von Willebrand factor and adhesion molecules) and inflammatory cytokine signaling promote platelet adhesion and coagulation activation in many thrombocytopenic settings, including cancer and critical illness [21,22]. Impaired fibrinolysis and coagulation system abnormalities like elevated factor VIII and a hypofibrinolytic profile (e.g., increased plasminogen activator inhibitor-1 and prolonged clot lysis time in liver disease) can favor thrombus persistence and propagation, further decoupling platelet count from thrombotic risk [12,14].

The dominant drivers vary by etiology: in cancer, tumor-driven hypercoagulability and procoagulant extracellular vesicles predominate [23]; in cirrhosis, parallel changes in pro- and anticoagulant pathways create a precarious rebalance [24]; in immune thrombocytopenia, immune activation, treatments (e.g., corticosteroids, TPO-RAs, and IVIG), and patient comorbidities influence risk [25]; and in consumptive disorders such as TMA or sepsis-associated coagulopathy, microvascular thrombosis is integral to pathophysiology, making thrombocytopenia a marker of severity rather than protection [26,27,28,29,30,31,32,33].

Given this shared framework, antithrombotic decisions should integrate the clinical indication (e.g., VTE/CAT, atrial fibrillation, portal vein thrombosis, TMA, and HIT) with platelet-based safety thresholds, guideline recommendations, and real-world outcome data.

## 4. Anticoagulation by Clinical Indication in Thrombocytopenia

### 4.1. Venous Thromboembolism and Cancer-Associated Thrombosis (VTE/CAT)

CAT is a leading cause of morbidity and mortality in oncology patients, with venous thromboembolism (VTE) representing the second most common cause of death after cancer progression itself [34,35]. Malignancy confers a 4–7-fold increased risk of thrombosis compared with the general population, driven by tumor-related hypercoagulability, systemic inflammation, endothelial activation, and procoagulant extracellular vesicles [34]. Thrombocytopenia, arising from bone marrow infiltration, chemotherapy, or immune-mediated mechanisms, is present in approximately 20–50% of patients with acute CAT [36].

Importantly, thrombocytopenia does not protect against recurrent thrombosis in cancer. Prospective cohort studies demonstrate that even mild thrombocytopenia is associated with increased risks of both bleeding and recurrent VTE [34,36]. Consequently, anticoagulation remains essential but must be carefully adapted to platelet count and thrombotic severity.

The ISTH Scientific and Standardization Committee (SSC) [37] and EHA [38] provide the most widely adopted guidance for CAT management in thrombocytopenic patients. For patients with platelet counts ≥ 50 × 10^9^/L, full-dose therapeutic anticoagulation with low-molecular-weight heparin (LMWH) or direct oral anticoagulants (DOACs) is recommended without routine platelet transfusion. For platelet counts of 25–50 × 10^9^/L, treatment intensity is determined by thrombotic risk. High-risk features—such as symptomatic proximal deep vein thrombosis (DVT), pulmonary embolism (PE) with cardiopulmonary compromise, or extensive clot burden—justify full-dose anticoagulation supported by platelet transfusions to maintain counts ≥40–50 × 10^9^/L. Lower-risk events, including distal DVT, isolated subsegmental PE, and catheter-related thrombosis, may be managed with a 50% dose reduction or prophylactic-intensity anticoagulation. Anticoagulation is generally withheld when platelet counts fall below 25 × 10^9^/L, unless thrombosis is life-threatening. In such cases, individualized strategies incorporating platelet transfusion support or temporary mechanical protection (e.g., inferior vena cava filters) may be considered. Anticoagulation should be resumed promptly once platelet recovery occurs.

Regarding anticoagulant choice, LMWH remains the most extensively studied agent in CAT with thrombocytopenia due to its predictable pharmacokinetics and short half-life. DOACs are increasingly used and supported by randomized trials in CAT; however, patients with severe thrombocytopenia were largely excluded from these studies [39]. Vitamin K antagonists are generally discouraged due to drug–drug interactions, labile anticoagulation, and unreliable INR measurements in cancer-related inflammation. Ongoing prospective studies aim to compare platelet-adapted LMWH dosing strategies and may provide critical evidence to refine current recommendations.

Cancer patients with thrombocytopenia face substantial bleeding risks regardless of management strategy. In a systematic review of 1728 cancer patients with thrombocytopenia, major bleeding occurred at 4.45/100 patient-months (95% CI 2.80–7.06) with full-dose anticoagulation and 4.16/100 patient-months (95% CI 2.24–7.74) with modified doses [40].

When comparing anticoagulation strategies in hospitalized cancer patients with severe thrombocytopenia (platelets 20–50 × 10^9^/L), reduced-dose dalteparin (100 units/kg daily) resulted in an 8.6% bleeding incidence, similar to the 9.4% rate in comparator patients receiving standard doses (risk ratio 0.94, 95% CI 0.37–2.39, *p* = 0.607) [41]. Bleeding types included hematemesis, hematuria, severe epistaxis, upper GI hemorrhage, and postoperative wound site hemorrhage. In patients with hematologic malignancies and severe thrombocytopenia (<50 × 10^9^/L), clinically significant bleeding occurred in 27% of patients receiving anticoagulation versus only 3% when anticoagulation was held (IRR 10.1, 95% CI 1.5–432.6). Most bleeding events occurred within the first 30 days, at a median platelet count of 38 × 10^9^/L. Bleeding types included mucocutaneous (eight events), deep tissue (two events), pulmonary (two events), and retroperitoneal (one event) [42].

In cancer patients, recurrent venous thromboembolism remained a persistent concern. Full-dose anticoagulation resulted in recurrent VTE at 2.65/100 patient-months (95% CI 1.62–4.32), while modified doses yielded 3.51/100 patient-months (95% CI 1.00–12.39). Among hospitalized cancer patients with severe thrombocytopenia, new-onset VTE occurred in 5.7% receiving reduced-dose dalteparin versus 1.9% in those receiving standard doses (odds ratio 3.31, 95% CI 0.29–37.90, *p* = 0.556) [43].

The timing of thrombotic events showed distinct patterns. In hematologic malignancies, most recurrent VTE occurred after day 40 of severe thrombocytopenia, contrasting with the earlier occurrence of bleeding events (predominantly before day 31). Anticoagulation reduced recurrent VTE from 15% to 2% (IRR 0.17, 95% CI 0.0–1.51), though no deaths were attributed to either recurrent VTE or bleeding [42].

Cancer patients with severe thrombocytopenia (platelets < 50 × 10^9^/L) face substantially different risk margins. Even with dose modification, major bleeding rates remained high at 4.16–4.45/100 patient-months, while recurrent VTE occurred at 2.65–3.51/100 patient-months [40]. The similarity of these rates suggests that neither outcome dominates the risk–benefit calculation at a population level. However, granular examination reveals important distinctions: in hematologic malignancies with acute severe thrombocytopenia, continuing anticoagulation increased bleeding tenfold (27% vs. 3%, IRR 10.1), whereas withholding anticoagulation increased recurrent VTE 7.5-fold (15% vs. 2%). The differential temporal patterns—bleeding predominantly within 30 days versus VTE predominantly after 40 days—suggest that temporarily withholding anticoagulation during acute severe thrombocytopenia, then resuming at platelet recovery, may optimize the risk–benefit balance by avoiding the highest-risk bleeding period while still providing thrombotic protection during the higher-risk VTE period [42].

### 4.2. Atrial Fibrillation

Patients with atrial fibrillation and thrombocytopenia were found to be at an increased risk of bleeding compared to those with normal platelet counts. In the largest propensity-matched cohort, the cumulative incidence of clinically relevant bleeding at one year was 18.3% (95% CI, 13.6–22.9) in thrombocytopenic patients versus 13.2% (95% CI, 10.9–15.6) in controls (*p* < 0.001). Major bleeding occurred in 10.1% (95% CI, 6.5–13.7) of thrombocytopenic patients compared to 5.2% (95% CI, 3.7–6.8) of controls (*p* < 0.001). The study recorded 90 major bleeding events and 122 clinically relevant non-major bleeding events across both groups [44]. However, when using reduced-dose LMWH, the bleeding risk differential narrowed substantially. Janion-Sadowska et al. [45] reported similar rates between thrombocytopenic and non-thrombocytopenic patients: major bleeding occurred at 1.8%/year versus 2.7%/year (*p* = 0.49), and clinically relevant non-major bleeding at 1.5%/year versus 1.1%/year (*p* = 0.74). Age emerged as the only significant predictor of bleeding in thrombocytopenic patients (hazard ratio 1.1, 95% CI 1.0–1.3, *p* = 0.04) [45].

In patients treated with antivitamin K with moderate thrombocytopenia, minor bleeding occurred three times more frequently than in normal patients (IRR 3.03, 95% CI 1.57–5.60), while major bleeding showed a trend toward increased risk (IRR 1.48, 95% CI 0.44–3.98). Higher INR values were associated with bleeding events [43].

Thrombotic protection remained generally effective across anticoagulation strategies. In atrial fibrillation, reduced-dose non-vitamin K drugs (NOAC) provided stroke prevention comparable to standard dosing in patients without thrombocytopenia, with stroke/TIA rates of 1.8%/year versus 1.5%/year (*p* = 0.8). Mortality rates were also similar (1.06%/year vs. 1.11%/year, *p* = 0.96). Patients taking warfarin who had thrombocytopenia derived similar benefits against thrombotic events as those with normal platelet counts. (IRR 0.807, 95% CI 0.09–3.43) [45].

Atrial fibrillation patients with mild-to-moderate thrombocytopenia (50–100 × 10^9^/L) are often considered the most permissive scenario in which anticoagulation may be continued; however, it must be emphasized that major guidelines and consensus documents do not currently recommend a reduced-dose DOAC strategy specifically because of thrombocytopenia, and the supporting evidence remains extremely limited. The available data are largely observational and derived from selected cohorts. In these reports, use of reduced-dose DOACs was associated with bleeding rates in thrombocytopenic patients approaching those of patients with normal platelet counts, while stroke outcomes appeared similar, although these findings should be interpreted cautiously given the non-randomized design and limited event numbers [45]. In contrast, thrombocytopenic AF patients treated with standard-dose regimens showed higher major bleeding rates (10.1% vs. 5.2% at one year) [44]. While some authors have proposed specific reduced-dose regimens (e.g., rivaroxaban 15 mg daily, dabigatran 110 mg twice daily, or apixaban 2.5 mg twice daily) as potentially safer in the 50–100 × 10^9^/L range, these dosing approaches should be regarded as hypothesis-generating rather than evidence-based standards and should not be presented as established practice.

Beyond platelet count, additional factors appear to modify bleeding risk. Age predicted bleeding in thrombocytopenic AF patients receiving DOACs [45], and in patients treated with vitamin K antagonists, higher INR values (particularly INR > 2.5) were associated with increased bleeding [43]. Comorbidities such as cancer, liver disease, and kidney disease were more prevalent in thrombocytopenic cohorts, but their independent contribution to bleeding risk cannot be reliably determined from the currently available data [44]. Although HAS-BLED scores were higher in thrombocytopenic AF patients (mean 2.0 vs. 1.0), reported bleeding rates with reduced-dose DOACs were similar to controls in one observational analysis; this observation is insufficient to conclude that dose reduction neutralizes baseline bleeding risk, but it supports further study [45].

Overall, while DOACs are increasingly used in thrombocytopenic patients across different clinical scenarios, the evidence base in atrial fibrillation with thrombocytopenia remains sparse and does not support firm recommendations. For example, Barlow et al. reviewed 27 articles totaling 104 patients and reported high rates of prevention of new/recurrent thrombosis with low reported bleeding; nevertheless, these data come from heterogeneous indications, small sample sizes, and observational designs, limiting generalizability to AF [46]. Accordingly, any consideration of reduced-dose DOAC therapy in AF with thrombocytopenia should be framed as exploratory, requiring individualized thrombotic and bleeding risk assessment, careful monitoring, and, whenever possible, management within structured institutional protocols or prospective studies.

In summary, in atrial fibrillation with thrombocytopenia, reduced-dose DOACs should not be considered a guideline-supported strategy, and any use should be regarded as individualized and exploratory, given the limited and predominantly observational nature of the available evidence [43,44,45,46].

### 4.3. Liver Disease and Portal Vein Thrombosis

Thrombocytopenia in cirrhosis reflects portal hypertension-related hypersplenism, reduced thrombopoietin synthesis, and bone marrow suppression. Historically regarded as a bleeding-predominant condition, cirrhosis is now understood as a state of rebalanced hemostasis, characterized by parallel deficiencies in procoagulant and anticoagulant pathways [24].

International hepatology guidelines emphasize that INR and platelet count alone do not reliably predict bleeding risk, and routine correction of these parameters prior to invasive procedures or anticoagulation is discouraged [47,48]. Portal vein thrombosis (PVT) is a common complication of cirrhosis and is associated with disease progression, portal hypertension, and reduced transplant eligibility.

Current evidence supports anticoagulation for recent, occlusive, or progressive PVT, particularly in liver transplant candidates, once esophageal varices have been adequately managed [3,47,48]. Multiple meta-analyses demonstrate that anticoagulation significantly improves portal vein recanalization rates and reduces thrombus progression, with emerging evidence of improved overall survival [49]. Importantly, when variceal bleeding risk is controlled through endoscopic ligation and/or non-selective beta-blockers, anticoagulation does not significantly increase major bleeding rates.

LMWH remains the preferred first-line anticoagulant in advanced cirrhosis due to stable pharmacokinetics and independence from INR. DOACs appear safe and effective in selected patients with preserved hepatic function (Child–Pugh A–B), whereas their use in Child–Pugh C cirrhosis remains contraindicated due to insufficient evidence and altered drug metabolism [47,48,50]. Table 1 summarizes the main studies and their key conclusions regarding anticoagulation, including direct oral anticoagulants (DOACs), in patients with liver disease.

Although data are limited, most hematology and hepatology guidelines suggest initiating therapeutic anticoagulation when platelet counts exceed 50 × 10^9^/L, with cautious individualized decisions between 30 and 50 × 10^9^/L based on thrombotic urgency and bleeding risk [50]. Systematic correction of thrombocytopenia prior to anticoagulation is not recommended, although thrombopoietin receptor agonists may be considered in elective procedural settings.

### 4.4. Immune Thrombocytopenia (ITP)

Immune thrombocytopenia is characterized by immune-mediated platelet destruction and impaired platelet production. Despite low platelet counts, ITP is paradoxically associated with an increased incidence of venous and arterial thromboembolic events, estimated at 1.5–2.5 times that of the general population [55]. Thrombotic risk is influenced by patient-related factors (such as age and cardiovascular comorbidities), disease-related mechanisms (platelet activation, microparticles, and hypofibrinolysis), and treatment-related exposures (corticosteroids, splenectomy, and thrombopoietin receptor agonists).

Primary and secondary ITP share core pathogenic mechanisms; however, secondary forms—associated with autoimmune disease, infection, or lymphoproliferative disorders—may exhibit additional thrombotic drivers and higher bleeding susceptibility [56]. Thrombosis in ITP may occur even at platelet counts lower than 50 × 10^9^/L, underscoring the need for individualized anticoagulation strategies.

Most expert recommendations support full-dose therapeutic anticoagulation when platelet counts are equal to or higher than 50 × 10^9^/L, provided there is no active bleeding [6,8,55,56]. Below this threshold, dose reduction or temporary interruption is considered based on bleeding phenotype and thrombotic severity. Importantly, ITP-directed therapies, such as intravenous immunoglobulin (IVIG) and corticosteroids, may be used acutely to increase platelet counts and facilitate safe anticoagulation initiation or resumption [6,7,8].

In cases of major bleeding, standard ITP emergency management—including platelet transfusions, IVIG, and high-dose corticosteroids—should be implemented, with careful reassessment of thrombotic risk once hemostasis is achieved. Secondary ITP may require control of the underlying disorder to achieve sustained platelet recovery [6,8,56].

### 4.5. Thrombotic Microangiopathies and Consumptive Thrombocytopenia

The incidence of thrombosis in patients with TMA and thrombocytopenia ranged from 18% to 51% across 10 studies encompassing 38,000 patients, with variation primarily reflecting disease context and detection methodology rather than contradictory findings. The highest incidence (51%) occurred in critically ill ICU patients with acute TMA, manifesting early (median 7 days from treatment initiation) as macrovascular thrombosis, including cerebral artery thrombosis in 13% and deep vein thrombosis in 38% of patients [57]. In complement-mediated TMA, venous thromboembolism affected 18.2% of patients at a rate of 5.6 events per 100 patient-years, occurring later (median 4.9 months) and predominantly provoked by catheters or hospitalizations [58]. For thrombotic thrombocytopenic purpura (TTP) survivors, cumulative relapse rates—representing recurrent thrombotic episodes—reached 18% at 4 years and 36% at 10 years. Critical risk factors included undetectable ADAMTS13 activity (OR 7.33), cardiac TMA involvement (OR 3.46 for thrombosis, OR 5.96 for mortality), and iatrogenic factors, with central venous catheters accounting for 50–62% of thrombotic events [57]. Notably, 80% of thrombotic events in complement-mediated TMA occurred after platelet count recovery above 150 × 10^9^/L, indicating thrombocytopenia does not protect against thrombosis and systemic thromboprophylaxis may be warranted once platelets recover [58]. Mortality directly attributable to thrombotic complications varied from 0% in anticoagulated complement-mediated TMA patients to 33% in drug-induced thrombotic thrombocytopenic purpura (TTP) [58], with plasma exchange reducing mortality by approximately 50% compared to alternative treatments [59].

Anticoagulation strategies in these settings are disease-specific and often adjunctive to definitive therapies (e.g., caplacizumab, emicizumab, plasma exchange, immunosuppression, and infection control). Routine anticoagulation is generally not indicated outside specific contexts and should be guided by specialist expertise [27,28,60]. Antiplatelet agents (aspirin and dipyridamole) showed a non-significant mortality benefit in acute TTP (2.8% vs. 13.5%), while ticlopidine maintenance therapy significantly reduced relapse rates (6.25% vs. 21.4%, *p* = 0.0182). Despite theoretical concerns about bleeding with anticoagulant and antiplatelet therapies in TMA/TTP, the observed bleeding rates varied by agent and context. In the Italian trial, bleeding did not worsen in patients receiving aspirin and dipyridamole, providing reassurance about acute antiplatelet use. The mucocutaneous bleeding represents a manageable adverse effect given the substantial reduction in TTP-related complications, suggesting an acceptable benefit-risk profile in the context of life-threatening TMA/TTP [61].

Caplacizumab is an anti-von Willebrand factor (vWF) nanobody that binds the vWF A1 domain, thereby blocking vWF–platelet (GPIb) interaction and preventing platelet adhesion in iTTP [62]. It is recommended as first-line treatment in iTTP [63,64]. Because it induces a defect in primary hemostasis, caplacizumab can increase bleeding risk, particularly when combined with antiplatelet or anticoagulant drugs [62,65,66]. To date, there are no clear, standardized guidelines on how to manage concomitant anticoagulation or antiplatelet therapy during caplacizumab treatment, so decisions are individualized.

In general, it could be suggested that anticoagulant or antiplatelet drug therapy be introduced as secondary prophylaxis in patients with platelet counts higher than 50 × 10^9^/L [61,63,64,67].

### 4.6. Heparin-Induced Thrombocytopenia (HIT)

HIT is a distinct immune-mediated, strongly prothrombotic adverse drug reaction driven by platelet-activating antibodies against platelet factor 4 (PF4)–heparin complexes. Unlike most other thrombocytopenic states, suspected HIT mandates immediate discontinuation of all heparin products and prompt initiation of a non-heparin anticoagulant, even when thrombosis has not yet been documented [68]. Diagnosis and initial management are guided by pretest probability assessment (e.g., the 4Ts score), followed by immunoassays and, when available, functional assays. In patients with intermediate or high clinical probability, ASH guidance supports empiric initiation of a non-heparin anticoagulant while awaiting confirmatory testing; during this suspected phase, intensity should be individualized, with prophylactic-intensity dosing considered only in patients at high bleeding risk, whereas therapeutic-intensity dosing is recommended when bleeding risk is not high and for high-probability cases [68].

Real-world outcome data comparing non-heparin anticoagulants remain largely observational. In a systematic review and meta-analysis of 92 studies (4698 patients), pooled outcomes were broadly comparable across agents; fondaparinux and DOACs showed favorable effectiveness and low major bleeding rates in selected populations, supporting them as practical alternatives where intravenous agents are unavailable or less suitable [69]. However, once HIT is diagnosed/confirmed (acute HIT), anticoagulation should be initiated or continued at therapeutic-intensity dosing regardless of the platelet count, given the markedly prothrombotic nature of the condition [68].

Recommended treatment options for acute HIT include non-heparin anticoagulants, with argatroban and danaparoid representing the classically labeled agents in this setting, and other parenteral options, such as bivalirudin and fondaparinux. DOACs may be considered in clinically stable patients, applying standard contraindications for acute VTE while recognizing that the evidence is largely observational [68,69,70]. Vitamin K antagonists should be avoided until platelet recovery (typically ≥150 × 10^9^/L) because of the risk of limb gangrene and skin necrosis in acute HIT [68]. In patients with very low platelet counts (e.g., <50 × 10^9^/L), close clinical monitoring is warranted to enable early detection of severe bleeding, but therapeutic anticoagulation should not be down-titrated solely on the basis of thrombocytopenia once HIT is confirmed [70].

## 5. Antiplatelet Therapy in Thrombocytopenic Patients

### 5.1. General Principles and Clinical Challenges

Antiplatelet therapy in thrombocytopenic patients represents a distinct and often more complex challenge than anticoagulation, as it directly interferes with primary hemostasis in the setting of quantitative platelet deficiency. Unlike anticoagulants—where thrombin generation may remain preserved—antiplatelet agents impair platelet adhesion, activation, and aggregation, thereby amplifying bleeding risk even at moderate degrees of thrombocytopenia.

Patients with thrombocytopenia have been systematically excluded from randomized clinical trials evaluating aspirin, P2Y12 receptor antagonists, and dual antiplatelet therapy (DAPT). Consequently, evidence is derived primarily from retrospective cardiovascular cohorts, subgroup analyses, and expert consensus statements from cardiology and hematology societies. This limitation necessitates cautious extrapolation and reinforces the importance of individualized decision-making.

Key determinants guiding antiplatelet use include: platelet count and trend, indication (primary vs. secondary prevention), ischemic risk (acute coronary syndrome, recent stent implantation), bleeding phenotype, and comorbidities. Table 2 describes platelet-based safety thresholds for antiplatelet therapy in thrombocytopenic patients.

Antiplatelet therapy provides clear ischemic protection in high-risk arterial settings (e.g., acute coronary syndrome and recent stent implantation), where platelet-driven thrombosis is a dominant mechanism. Potential advantages include prevention of stent thrombosis and recurrent myocardial infarction and the feasibility of de-escalation strategies (shortened DAPT and early transition to monotherapy) facilitated by contemporary drug-eluting stents. However, in thrombocytopenia, antiplatelet agents directly impair primary hemostasis and may disproportionately increase bleeding risk, particularly gastrointestinal and intracranial bleeding, and especially when combined with anticoagulation. Additional disadvantages include limited evidence (systematic exclusion from trials), drug-specific bleeding gradients (higher bleeding risk with ticagrelor/prasugrel vs. clopidogrel), variability in platelet counts over time, and amplified risk in the presence of high-risk lesions (e.g., varices) or concomitant thrombocytopenia etiologies with intrinsic platelet dysfunction. Practical mitigation includes strict indication review (primary vs. secondary prevention), preference for single antiplatelet therapy whenever feasible, use of clopidogrel as the preferred P2Y12 inhibitor in many thrombocytopenic contexts, shortest effective DAPT duration, gastroprotection when appropriate, and multidisciplinary decision-making integrating platelet trend and bleeding phenotype [71,72,73,74,75].

### 5.2. Aspirin in Thrombocytopenia

Low-dose aspirin (75–100 mg/day) remains a cornerstone of secondary prevention in atherosclerotic cardiovascular disease. Observational data suggest that aspirin may be used relatively safely in thrombocytopenic patients with platelet counts equal to or higher than 50 × 10^9^/L, provided there is no active bleeding or high-risk lesion [71,72].

In platelet counts between 30 and 50 × 10^9^/L, aspirin may be considered only when ischemic risk clearly outweighs bleeding risk, such as in recent myocardial infarction or symptomatic coronary artery disease. Below 30 × 10^9^/L, aspirin is generally contraindicated due to an unacceptable bleeding risk, particularly gastrointestinal and intracranial hemorrhage.

Importantly, aspirin use in immune thrombocytopenia deserves special consideration. Despite low platelet counts, ITP platelets are often hyperreactive; however, aspirin-induced inhibition of thromboxane A_2_ may critically impair residual platelet function. Therefore, aspirin should be avoided in ITP unless there is a compelling secondary prevention indication and platelet counts can be maintained above safety thresholds.

### 5.3. P2Y12 Receptor Antagonists

Among P2Y12 inhibitors, clopidogrel is generally preferred in thrombocytopenic patients due to its comparatively lower bleeding risk. In contrast, more potent agents such as ticagrelor and prasugrel are associated with significantly higher rates of major bleeding and are typically avoided when platelet counts are lower than 50 × 10^9^/L [73,74,75].

Current expert consensus supports clopidogrel monotherapy when platelet counts are ≥50 × 10^9^/L and ischemic risk is high and recommends avoidance of P2Y12 inhibitors when platelet counts are below 30 × 10^9^/L. Patients with fluctuating platelet counts or concomitant anticoagulation should be followed closely. Pharmacodynamic variability, drug–drug interactions, and altered absorption in critically ill or cirrhotic patients further complicate P2Y12 inhibitor use and warrant close monitoring too.

### 5.4. Dual Antiplatelet Therapy (DAPT)

DAPT is associated with a substantial increase in bleeding risk in thrombocytopenic patients and should be reserved for scenarios where ischemic benefit is unequivocal, such as acute coronary syndromes or recent percutaneous coronary intervention (PCI) with high-risk coronary anatomy.

Based on available evidence and expert opinion, in patients with platelets equal to or higher than 100 × 10^9^/L, standard DAPT may be used following general cardiology guidelines. In case of platelet counts from 50 to 100 × 10^9^/L, aspirin plus clopidogrel should be considered for the shortest feasible duration (often 1 month), followed by de-escalation to monotherapy. If platelet counts are lower than 50 × 10^9^/L, DAPT should generally be avoided; alternative strategies such as single antiplatelet therapy or deferred PCI should be discussed within a multidisciplinary team [73,74,75].

New-generation drug-eluting stents enabling abbreviated DAPT durations have partially mitigated bleeding risk but do not eliminate the need for careful platelet-based stratification [73,74,75].

## 6. Integrated Risk–Benefit Synthesis and Platelet-Based Thresholds

The evidence demonstrates that anticoagulation safety in thrombocytopenia depends critically on three contextual factors: underlying indication, platelet count threshold, and anticoagulant selection.

### 6.1. Dose–Response and Threshold Effects

The relationship between platelet count and bleeding risk appears non-linear with critical thresholds (Table 3 and Table 4). At platelets 50–100 × 10^9^/L, reduced-dose NOACs yielded bleeding rates of 1.5–1.8%/year for major bleeding [45]. At 20–50 × 10^9^/L, reduced-dose LMWH resulted in 8.6% bleeding incidence [41]. Below 50 × 10^9^/L with full anticoagulation, bleeding occurred in 27% of patients. This approximate doubling of bleeding risk with each halving of the platelet count range suggests an exponential rather than linear relationship. The median platelet counts at bleeding of 38 × 10^9^/L provide an empirical threshold below which bleeding risk may become prohibitive [42]. Supporting this, the protocol of withholding anticoagulation below 20 × 10^9^/L and resuming at >50 × 10^9^/L represents an evidence-based approach that mirrors this threshold effect [41].

Methodological considerations substantially influence interpretation. Studies have examined cancer patients with severe thrombocytopenia, providing more detailed data on bleeding types, timing, and platelet count relationships. The propensity-matched cohort of 1075 AF patients offers the strongest evidence for this population due to its size and matching methodology [44]. The systematic review of 1728 cancer patients noted a serious risk of bias across all included studies, limiting confidence in pooled estimates [40].

The single-center nature of several studies [44] and predominance of hematologic malignancies in cancer cohorts (90%) [40] limit generalizability to solid tumor patients or broader healthcare settings. The mean follow-up of 55 months in the Janion-Sadowska study provides longer-term safety data than most other investigations [41].

### 6.2. Practical Recommendations and Integrated Decision Algorithm

**Figure 2:**
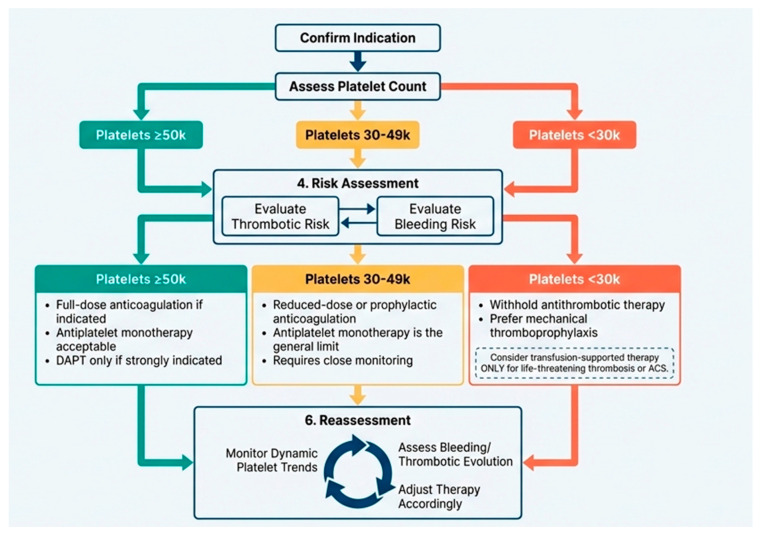
Integrated decision algorithm for antithrombotic therapy in thrombocytopenia. Integrated clinical decision algorithm for anticoagulation and antiplatelet therapy in patients with thrombocytopenia. Management is guided by platelet count, thrombotic risk, and bleeding risk, emphasizing individualized and dynamic reassessment. Figure was generated by the authors using NotebookLM (Google LLC, Mountain View, CA, USA).

Given the absence of randomized data, management of antithrombotic or antiplatelet therapy in thrombocytopenia should follow a structured, iterative, and multidisciplinary approach. Collaboration between hematologic and cardiovascular expertise should be based on an integrated, individualized assessment. Therapeutic decisions must consider three core domains: platelet count, thrombotic or ischemic risk, and bleeding risk. Platelet count is commonly stratified into three clinically relevant categories, namely ≥50 × 10^9^/L, 30–49 × 10^9^/L, and <30 × 10^9^/L, each associated with different safety profiles for antithrombotic therapy. Figure 2 presents a schematic overview of the proposed approach.

Assessment of thrombotic or ischemic risk is essential and should account for both the acuity and severity of the underlying condition. Patients at high risk include those with acute venous thromboembolism, massive pulmonary embolism, acute coronary syndromes, recent percutaneous coronary intervention, or mechanical heart valves. Intermediate-risk scenarios encompass distal deep vein thrombosis and stable coronary artery disease, whereas patients requiring antithrombotic therapy solely for primary prevention are generally considered low risk.

Bleeding risk must be evaluated alongside other factors, including a history of major bleeding, active bleeding, high-risk anatomical lesions such as untreated varices or intracranial pathology, and the concomitant use of multiple antithrombotic agents. These factors may significantly modify the net clinical benefit of anticoagulation or antiplatelet therapy, particularly in the setting of thrombocytopenia.

Based on this integrated assessment, patients with platelet counts ≥ 50 × 10^9^/L can generally receive full-dose therapeutic anticoagulation when clinically indicated. In this group, antiplatelet monotherapy is acceptable for secondary cardiovascular prevention, while dual antiplatelet therapy should be reserved for situations in which ischemic benefit clearly outweighs bleeding risk. For patients with platelet counts between 30 and 49 × 10^9^/L, anticoagulation strategies should be individualized, favoring reduced-dose or prophylactic-intensity regimens according to thrombotic risk. In this range, antiplatelet therapy is typically limited to monotherapy and only in carefully selected cases.

When platelet counts fall below 30 × 10^9^/L, antithrombotic therapy is usually withheld due to the high risk of bleeding. Exceptions may be considered in life-threatening situations, such as massive thrombosis or acute coronary syndromes, where short-term, transfusion-supported strategies may be justified within a multidisciplinary framework. Across all platelet strata, frequent reassessment is essential, as platelet counts, bleeding risk, and thrombotic burden may evolve rapidly over time, necessitating dynamic adjustment of therapeutic intensity.

## 7. Knowledge Gaps and Future Directions

Key evidence gaps persist because severe thrombocytopenia populations are underrepresented in randomized trials. Priority research areas include: (i) prospective studies validating platelet-adapted dosing and transfusion-supported strategies for acute high-risk VTE and arterial events; (ii) comparative effectiveness of abbreviated DAPT and early de-escalation in thrombocytopenic patients undergoing PCI; (iii) development of integrated risk models combining platelet count with bleeding phenotype, thrombotic acuity, and disease-specific prothrombotic drivers; and (iv) evaluation of biomarkers (e.g., thrombin generation, platelet activation and endothelial markers) to refine prediction beyond platelet thresholds alone. These efforts are essential to move from pragmatic, consensus-based thresholds toward precision, etiology-informed antithrombotic management.

## 8. Conclusions

Thrombocytopenia represents a complex and heterogeneous clinical condition in which bleeding and thrombosis frequently coexist. Contemporary evidence demonstrates that low platelet counts do not confer intrinsic protection against thrombotic events and should not, in isolation, preclude anticoagulation or antiplatelet therapy.

Management requires careful balancing of ischemic and bleeding risks, with platelet-based thresholds serving as pragmatic—but imperfect—guides. Disease-specific factors, particularly in cancer-associated thrombosis, immune thrombocytopenia, and advanced liver disease, critically influence therapeutic decisions.

Until high-quality prospective data become available, individualized, platelet-adapted strategies supported by multidisciplinary collaboration remain the cornerstone of optimal care. Structured algorithms, as proposed in this review, may help harmonize practice and improve outcomes in this vulnerable population.

## Figures and Tables

**Table 1:** Key studies evaluating anticoagulation in portal vein thrombosis (PVT) in patients with cirrhosis and thrombocytopenia.

Study	Clinical Context	N	Anticoagulant	Platelet Threshold	Thrombotic Outcomes	Bleeding Outcomes
Ng CH et al., 2021 [51]	Meta-analysis/network analysis: DOACs vs. LMWH vs. VKA in cirrhotic PVT	Series (variable)	DOACs vs. LMWH/VKA	Heterogeneous; not reported	↑ Complete recanalization with DOACs vs. VKA	Major bleeding ↔ DOACs vs. LMWH; similar/↓ vs. VKA
Guerrero A et al., 2023 [52]	IPD meta-analysis: cirrhosis + PVT (treated vs. untreated)	500	LMWH/VKA/DOAC vs. none	Heterogeneous; limited	↑ Recanalization (adj OR ~3.45); ↓ mortality (sHR 0.59)	No consistent ↑ in portal HTN-related bleeding; variceal bleeding not consistently ↑
Wang L et al., 2021 [53]	Meta-analysis of cohorts/trials: anticoagulation in cirrhotic PVT	1696	LMWH/VKA/DOAC	Often not defined	↑ Recanalization (RR ~2.6) and ↓ progression; earlier initiation better	Overall bleeding ↔ (pooled); heterogeneity across studies
Primignani M et al., 2022 [54]	Review + real-world series: DOAC use in cirrhotic PVT	Series (variable)	DOACs	Typically >30–50 × 10^9^/L in practice	Recanalization ↔/↑ vs. VKA; favorable tolerability in some cohorts	Major bleeding ↔/↓ vs. VKA; caution in Child–Pugh C

**Abbreviations:** PVT, portal vein thrombosis; DOACs, direct oral anticoagulants; LMWH, low-molecular-weight heparin; VKA, vitamin K antagonist; OR, odds ratio; RR, risk ratio; sHR, subdistribution hazard ratio. **Notes:** Most studies did not define strict platelet count thresholds for anticoagulation initiation. In clinical practice, anticoagulation was most commonly initiated at platelet counts ≥ 30–50 × 10^9^/L after assessment and management of portal hypertension-related bleeding risk. **Note.** Summary of key observational studies and meta-analyses evaluating anticoagulation in portal vein thrombosis (PVT) in patients with cirrhosis and thrombocytopenia. Across heterogeneous cohorts, anticoagulation—using low-molecular-weight heparin (LMWH), vitamin K antagonists (VKAs), or direct oral anticoagulants (DOACs)—is consistently associated with higher portal vein recanalization rates and reduced thrombus progression, without a significant increase in major bleeding when patients are appropriately selected. Platelet thresholds were variably reported, most commonly ≥30–50 × 10^9^/L in clinical practice. Caution is advised in advanced liver disease (Child–Pugh C).

**Table 2:** Platelet-based safety thresholds for antiplatelet therapy in thrombocytopenic patients.

Platelet Count (×10^9^/L)	Aspirin	Clopidogrel	Dual Antiplatelet Therapy (DAPT)
**>50**	Acceptable for secondary prevention	Acceptable; preferred P2Y12 inhibitor	Generally avoid; consider only if a strong ischemic indication
**30–50**	Consider only if ischemic benefit outweighs bleeding risk	Consider with caution; avoid potent P2Y12 inhibitors	Not recommended
**<30**	Contraindicated	Contraindicated	Contraindicated

**Footnote:** Potent P2Y12 inhibitors (ticagrelor, prasugrel) should be avoided in thrombocytopenic patients due to increased bleeding risk. Decisions should incorporate bleeding phenotype and indication (primary vs. secondary prevention).

**Table 3:** Anticoagulation strategies according to platelet count in thrombocytopenia.

Platelet Count (×10^9^/L)	LMWH	DOACs	Vitamin K Antagonists (VKAs)
≥50	Full-dose therapeutic anticoagulation	Full-dose acceptable (selected patients)	Use with caution
25–50	Dose-reduced or prophylactic-intensity; consider transfusion support if high thrombotic risk	Limited data; generally avoid	Discouraged
<25	Withhold unless life-threatening thrombosis	Contraindicated	Contraindicated

**Footnote:** LMWH remains the preferred anticoagulant in thrombocytopenic patients due to predictable pharmacokinetics and reversibility. DOACs may be considered in selected patients but are poorly studied at platelet counts < 50 ×10^9^/L.

**Table 4:** Venous thromboembolism (VTE) prophylaxis in hospitalized thrombocytopenic patients.

Platelet Count (×10^9^/L)	Recommended VTE Prophylaxis
**≥50**	Pharmacological prophylaxis (LMWH)
**30–50**	Individualized; consider reduced-dose LMWH
**<30**	Mechanical prophylaxis only

**Footnote:** Thrombocytopenia alone should not preclude pharmacological prophylaxis when platelet counts are ≥50 ×10^9^/L and no active bleeding is present.

## Data Availability

This article did not report any data.
